# 10-Year outcome of descemet stripping only in a patient with Fuchs endothelial dystrophy: a case report

**DOI:** 10.1177/25158414251359583

**Published:** 2025-08-30

**Authors:** Fuad Moayed, Friedrich Anton Steindor, Zaira Eleni Armeni, Markus Kohlhaas, Gerd Geerling

**Affiliations:** Department of Ophthalmology, Medical Faculty, University Hospital Düsseldorf, Moorenstraße 5, Düsseldorf 40225, Germany; Department of Ophthalmology, University Hospital Düsseldorf, Düsseldorf, Germany; Department of Ophthalmology, St. Johannes Hospital, Dortmund, Germany; Department of Ophthalmology, St. Johannes Hospital, Dortmund, Germany; Department of Ophthalmology, University Hospital Düsseldorf, Düsseldorf, Germany

**Keywords:** DMEK, DSO, DWEK, endothelial keratoplasty, FED

## Abstract

In recent years, descemet stripping only (DSO) has emerged as an alternative to descemet membrane endothelial keratoplasty (DMEK) in certain patients with Fuchs endothelial dystrophy (FED). We herein report the 10-year follow-up of a 77-year-old male patient after bilateral DSO. The patient initially underwent DSO on the right eye for circumscribed cornea guttata. Three weeks after DSO, the best-corrected visual acuity (BCVA) already increased from 0.5 logarithm of the Minimum Angle of Resolution (logMAR) [Endothelial Cell Density (ECD) 1667/mm^2^, Central Corneal Thickness (CCT) 583 µm] to 0.2 logMAR, and further improved to 0 logMAR 1 year after surgery (ECD 2213/mm^2^, CCT 567 µm). This excellent visual acuity remained stable over the following 5 years (ECD 1696/mm^2^, CCT 568 µm). Five years after the successful surgery on the right eye, DSO was also performed on the left eye by the same surgeon as FED progressed, with BCVA dropping to 0.5 logMAR (ECD unmeasurable, CCT 703 µm). However, this time, the treatment did not improve vision. Consequently, a DMEK was performed 7 months after DSO, which increased the BCVA to 0.1 logMAR. Ten years after successful DSO of the right eye, corneal guttata were observed, indicating de novo formation of a descemet membrane, and vision deteriorated again to 0.2 logMAR (ECD not measurable, CCT 641 µm). DMEK was also performed on the right eye ten years after successful DSO, which improved vision to 0.2 logMAR at one-year follow-up. This case suggests that DSO may be a temporary alternative to DMEK in FED, potentially providing excellent visual gain and good central endothelial cell density for nearly ten years. However, it may still fail due to long-term progression of the disease. It also highlights that the outcome may be limited by individual factors. Therefore, it is crucial to educate the patient about the limitations of DSO, both in short and long term. Nevertheless, if DSO fails, endothelial keratoplasty can still be successfully performed.

## Introduction

Fuchs endothelial dystrophy (FED) is a hereditary disorder characterized by endothelial cell loss and dysfunction, leading to corneal edema, visual impairment, and in advanced stages, corneal opacity and vascularization. The condition often progresses slowly over decades, affecting predominantly older individuals, with a higher incidence in females.^1,2^

For decades, the primary surgical treatment for FED was penetrating keratoplasty. However, this changed when Melles first described descemet membrane endothelial keratoplasty (DMEK) in 2006.^3^ Over time endothelial keratoplasty techniques evolved and the approach to treating FED has undergone a major shift. Endothelial Keratoplasty (EK) focuses specifically on replacing the dysfunctional endothelium rather than the entire cornea, preserving the structural integrity of the corneal stroma. Especially DMEK is considered the gold standard due to its superior postoperative recovery, excellent visual outcomes, and lower rates of complications, such as graft rejection and immune reactions.^4–9^

Today, DMEK is also used in difficult cases, for example, after vitrectomy or even previous penetrating keratoplasty.^10–12^ However, even if the risk of immune reaction is significantly reduced, long-term topical steroid therapy is still often recommended for prophylaxis and carries the risk of a steroid-induced intraocular pressure increase.^13^

Following reports of spontaneous resolution of corneal edema and good visual outcomes after iatrogenic descemetorhexis during phacoemulsification or incomplete DMEK graft attachment^14–16^ selective stripping of the central 4–5 mm of DM without a tissue replacement (DSO or Descemetorhexis Without Endothelial Keratoplasty (DWEK)) was used in some cases with limited (i.e., central) FED.^17–19^ Due to the migration (and potentially proliferation) of endothelial cells into the center, the corneal edema resolved and visual acuity improved. Ruhe et al.^19^ reported migration-related reendothelialization and a good functional outcome in a 75-year-old patient with no prior corneal disease, but a 5 mm descemetolysis after phacoemulsification, as was also reported by Moloney et al.^20^ in two cases. Multiple reports of DSO with short-term follow-up have shown good visual rehabilitation,^17,18,21,22^ whereas the longest published follow-up is limited to 5 years and very few cases.^23,24^ Here, we report the 10 year follow-up of a case of bilateral DSO in FED first published in 2017.^25^ This case is unique, offering insight into the long-term outcomes of DSO over nearly a decade and its potential as a viable alternative to DMEK for selected patients with FED.

## Case presentation

**Figure 1.**
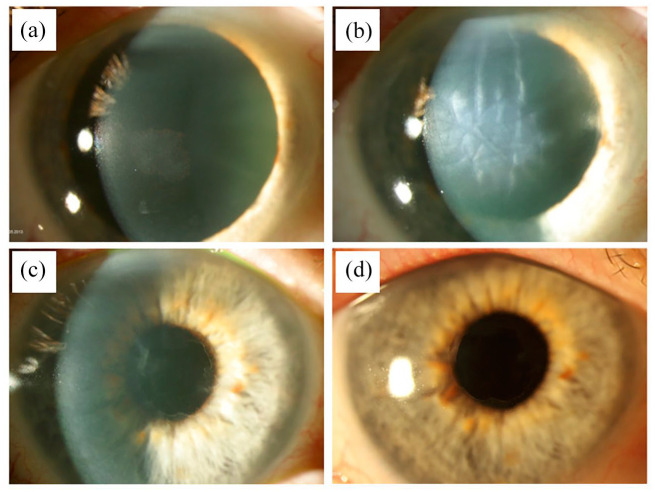
(a) Central endothelial guttae in the right eye preoperative and cataract. BCVA 0.5 logMAR. (b) 1 day postoperative after DSO. Corneal edema, descemet folds and centered intraocular lens. (c) 3 weeks follow-up after DSO. Corneal transparency improved and a well-demarcated central Descemet’s membrane defect on slit-lamp examination. BCVA 0.2 logMAR. (d) 5-year follow-up after DSO. Cornea is clear without any signs of deterioration. BCVA 0 logMAR. BCVA, best-corrected visual acuity; DMEK, descemet membrane endothelial keratoplasty; DSO, descemet stripping only.

A 77-year-old male patient presented to our department in 2012 with right-sided photophobia and morning visual loss for approximately 2 years. The patient had a history of primary open angle glaucoma since 2006, which was treated with dorzolamide, timolol and tafluprost eyedrops. The family history was negative for any hereditary eye diseases, including Fuchs’ endothelial dystrophy (FED), glaucoma, or other corneal pathologies. No significant genetic conditions were reported. BCVA in logMAR was 0.5 in the right eye and 0.1 in the left eye, with intraocular pressures of 15 and 11 mmHg, respectively. Slit-lamp examination revealed localised central endothelial guttae predominantly in the right eye (Figure 1(a)), as well as bilateral cataracts. CCT and central endothelial cell count using Oculus Pentacam^®^ HR (OCULUS GmbH, Wetzlar, Germany) and EM-3000^®^ specular microscope (Tomey Corporation, Nagoya, Japan) were 583 μm, 1667/mm^2^ in the right and 571 μm, 1905/mm^2^ in the left eye. Anterior segment findings were otherwise normal. Funduscopy revealed normal findings except for bilateral glaucomatous optic disc cupping with vertical cup-to-disc ratio of 0,8 right and 0,9 on the left eye (Supplemental Material).

Due to the limited area of the central guttae we performed a central descemetectomy of 3 mm diameter with simultaneous phacoemulsification and posterior chamber lens implantation in the right eye. DSO was performed under anterior chamber air fill using a Price hook for a 360° scoring and DM peeling. After uneventful surgery, central corneal stromal edema was present on the first postoperative day, limiting BCVA to 0.5 logMAR (Figure 1(b)). Postoperative medication included dexamethasone 1 mg/ml eye drops five times daily and ofloxacin 3 mg/ml eye drops five times daily. No RhOCK inhibitor was used.

**Figure 2.**
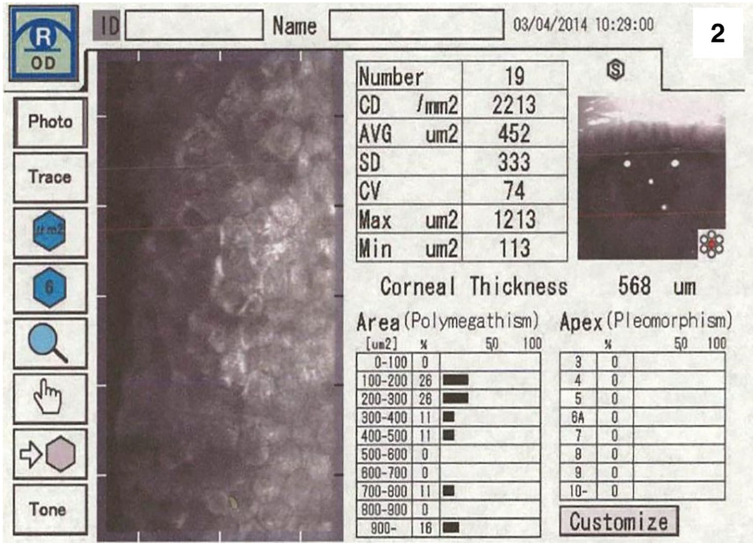
Specular microscopy at 1-year follow-up of the right eye. Slit lamp examination showed confluent endothelial cells in the area of DSO. DSO, descemet stripping only.

Three weeks after surgery, BCVA improved to 0.2 logMAR on the right eye with markedly improved corneal transparency with a well-demarcated central Descemet’s membrane defect on slit-lamp examination (Figure 1(c)). BCVA increased further in the following course to 0.1 logMAR at three and 0 logMAR at 1-year follow-up. This was associated with resolution of photophobia and corneal edema. Specular microscopy at 1-year follow-up of the right eye showed confluent endothelial cells in the area of DSO and an ECD 2213/mm^2^ (Figure 2) with a corneal thickness of 567 µm. At 5 years after DSO of 3 mm diameter CCT remained at 568 µm and the ECD was 1696/mm^2^, BCVA was still at 0 logMAR (Figure 1(d)).

However, 4 years after cataract surgery in the left BCVA deteriorated to 0.5 logMAR with pronounced corneal guttae and photophobia. ECD could not be measured successfully, but central corneal thickness was 703 µm. Subsequently, DSO was also performed in the left eye as well. However, since central corneal edema persisted in this left eye with a CCT of 787 µm and BCVA remained limited to 0.4 logMAR BCVA postoperatively, a DMEK was performed 7 months later as previously described.^7^ This resulted in an improvement of BCVA with 0.1 logMAR which was maintained for remaining follow-up.

Ten years after the initial, successful DSO, he reported a recurrent loss of vision for approximately 3 months in the right eye. BCVA was 0.2 logMAR in his right eye with a CCT of 641 µm. Although ECD was no longer measurable on the right eye slit-lamp examination showed pronounced corneal guttae in the area where DM had previously been removed. The clinical finding was supported by a preoperative specular microscopy image showing distinct central guttae in the area of the previous descemet stripping (Figure 4). This observation may represent the first documented case of central guttae formation following an initially successful DSO. A DMEK procedure was subsequently performed in this right eye using a standard technique including stripping of the central DM. During the procedure a membrane was removed from the central area of the previous Descemet stripping, although increased adhesions had to be overcome. Unfortunately, the tissue was not sent for histological analysis and no slit-lamp or specular microscopy images were available after the rescue DMEK. Thus, we have no histological proof of regeneration of Descemet Membrane after DSO. Following successful DMEK surgery in the right eye, BCVA improved to 0.2 logMAR after 1 year. In the left eye, 3 years after DMEK, BCVA remained at 0.1 logMAR with a CCT of 528 µm and ECD of 735/mm^2^.

## Timeline of clinical events

**2012 – Initial presentation:**

• Patient presents with right-sided photophobia and morning visual loss for approximately 2 years.
• Right eye:
   ○ BCVA in logMAR: 0.5
   ○ Slit-lamp examination: Localized central endothelial guttae in right eye (Figure 1(a)), cataract.
   ○ CCT and endothelial cell count:
    ■ Right eye: 583 μm, 1667/mm²

• Left eye:
   ○ BCVA in logMAR: 0.1
   ○ Slit-lamp examination: Clear cornea without endothelial guttae, cataract.
   ○ CCT and endothelial cell count:
    ■ Left eye: 571 μm, 1905/mm^2^

**2012 – Right eye surgery:**

- Procedure: Central descemetectomy (3 mm diameter) with simultaneous phacoemulsification and posterior chamber lens implantation. DSO performed under anterior chamber air fill with Price hook for 360° scoring and DM peeling.
- Postoperative BCVA in logMAR: 0.5 (on day 1), central corneal stromal edema present (Figure 1(b))

**2012 – 3 weeks postsurgery after DSO (right eye):**

- BCVA improved to 0.2 logMAR
- Slit-lamp examination: Markedly improved corneal transparency, central Descemet membrane defect (Figure 1(c))

**2013 – 1-year follow-up after DSO (right eye):**

- BCVA in logMAR: 0
- Specular microscopy: Confluent endothelial cells in area of DSO, ECD: 2213/mm², CCT: 567 μm (Figure 2)
- Resolution of photophobia and corneal edema

**2015 – Left eye surgery**

- Preoperative BCVA in logMAR: 0.2
- Procedure: phacoemulsification and posterior chamber lens implantation.
- 1 day postoperative BCVA in logMAR: 0.4

**2017 – 5-year follow-up after DSO (right eye):**

- BCVA in logMAR: 0
- Slit-lamp examination: Clear cornea with confluent endothelial cells in the area of previous DSO (Figure 1(d))
- ECD: 1696/mm², CCT: 568 μm

**2019 – 7-year follow up after DSO (right eye):**

**Figure 3.**
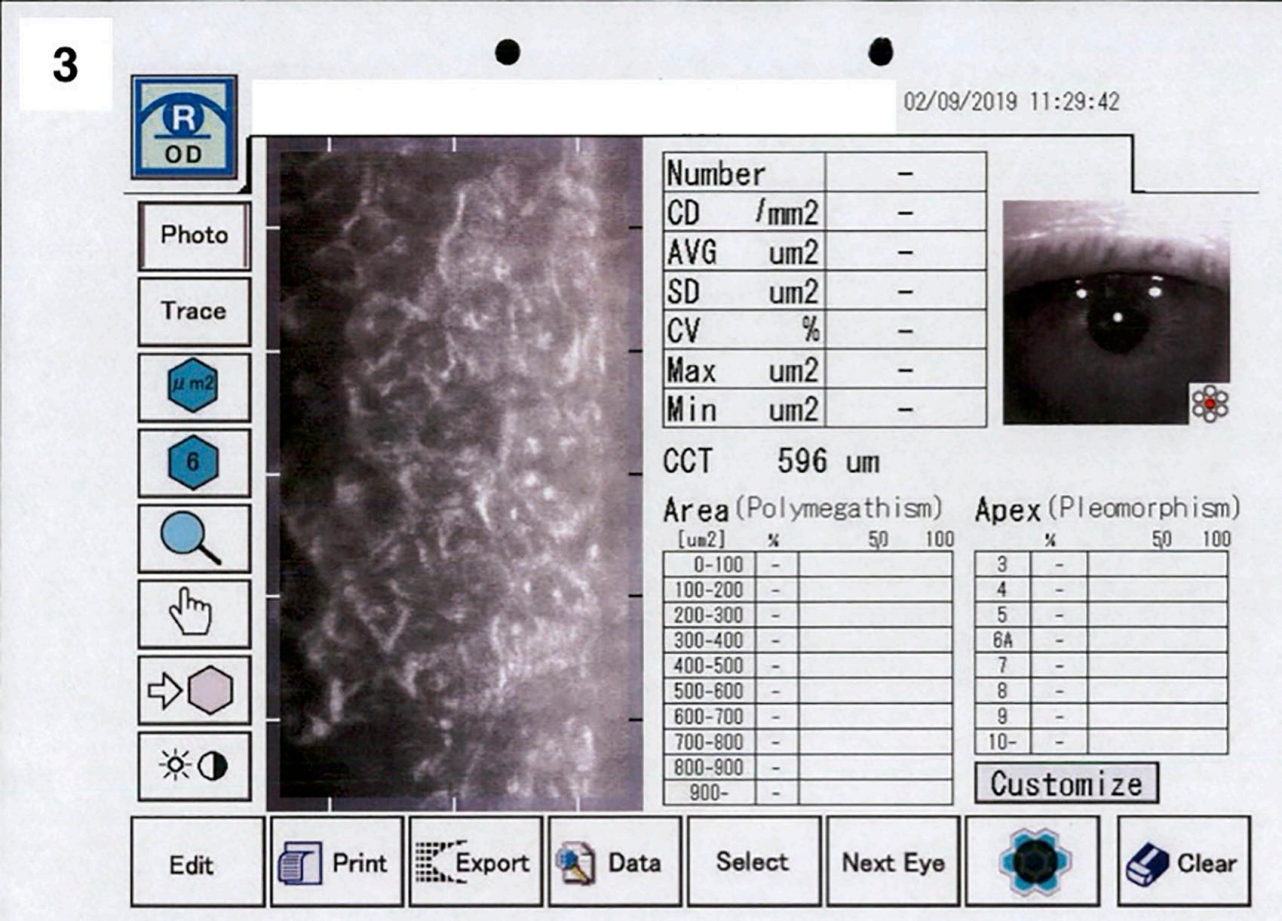
Specular microscopy of the right eye at 7-year follow-up showing central guttae in the area of the previous descemet stripping. CCT was 596 µm and ECD was not measurable.

- BCVA in logMAR: 0.2
- Specular microscopy: right eye showed central guttae with CCT of 596 µm and unmeasurable ECD (Figure 3)

**2019 – Left eye surgery:**

- BCVA deterioration to 0.5 logMAR
- Slit-lamp examination: pronounced corneal guttae and photophobia
- CCT: 703 μm
- DSO performed by same surgeon
- Procedure: Central descemetectomy (3 mm diameter). DSO performed under anterior chamber air fill with Price hook for 360° scoring and DM peeling

**2020 – 6-months follow-up after DSO (left eye):**

- BCVA in logMAR: 0.4 (left eye)
- Persistent central corneal edema (CCT: 787 μm),
- DMEK performed in left eye
- Post-DMEK BCVA in logMAR: 0.1 (left eye)

**2023 – 10-year follow-up after DSO (right eye):**

**Figure 4.**
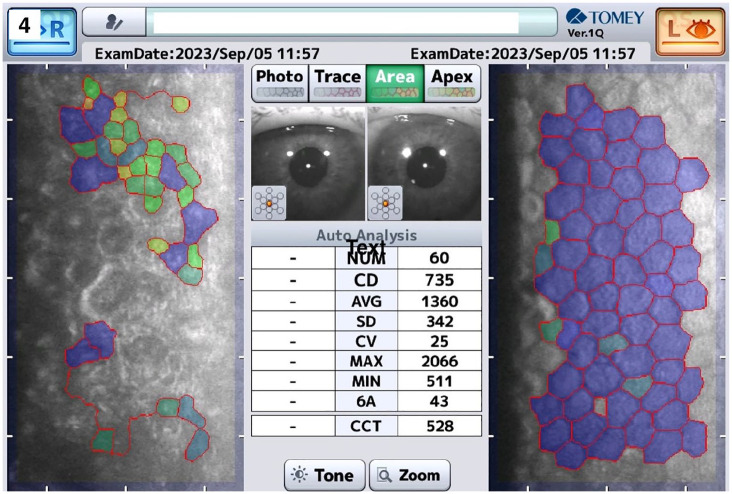
Specular microscopy of both eyes before performing DMEK 10 years after initial DSO in the right eye. Central guttae are present in the area of the previous Descemet stripping, with unmeasurable CCT and ECD. In contrast, the left eye (after DMEK) shows a confluent endothelial layer, with a central corneal thickness of 528 µm and an ECD of 735 cells/mm^2^. DMEK, descemet membrane endothelial keratoplasty; DSO, descemet stripping only.

- Recurrent visual loss in the right eye for 3 months
- BCVA in logMAR: 0.2
- CCT: 641 μm
- Slit-lamp examination: pronounced corneal guttae in area of previous DM removal
- Specular microscopy: right eye showed central guttae with unmeasurable CCT and ECD (Figure 4)
- DMEK performed in right eye

**2023 – 3-year follow-up after rescue DMEK (left eye):**

- BCVA in logMAR: 0.1
- CCT: 552 µm
- Slit-lamp examination: Cornea clear, graft well-positioned and attached, no visible signs of edema.
- Specular microscopy: left eye showed a confluent endothelium with CCT of 528 µm and ECD of 735 cells/mm² (Figure 4)

**2024 – 1-year follow-up after rescue DMEK (right eye):**

- BCVA in logMAR: 0.2
- Slit-lamp examination: Cornea clear, graft well-positioned and attached, no visible signs of edema.

## Discussion

The corneal endothelium is considered postmitotic, making spontaneous regeneration after loss of endothelial cells unlikely.^26^ Nevertheless, numerous publications now have reported clearing of the cornea after DSO, suggesting that this procedure may facilitate some endothelial healing.^24,27,28^ The case presented here confirms that areas of central endothelial changes in FED can be successfully managed by descemetectomy, resulting in spontaneous reendothelialization and a 10-year sustained visual improvement. The BCVA after 10 years was still 0.2 logMAR, which is only slightly worse than mean BCVA achieved after successful DMEK and still better than prior to DSO in this eye.^7,29^ However, the patient had noted this relatively minor recurrent loss of vision and secondary DMEK surgery was performed, indicating that visual quality after DSO can be considered excellent. In his fellow left eye however, DSO remained unsuccessful and an early rescue DMEK had to be performed.

Despite the promising results in this case, DSO’s outcomes can vary significantly based on patient-specific factors, such as the size of the descemetorhexis, age, initial endothelial cell density, the localization and extent of endothelial damage (central only or entire DM), and previous surgical interventions. However, Davies et al. found that factors like age, pachymetry, and ECD did not have a statistically significant impact on DSO outcomes. Instead, their findings emphasized the importance of surgical technique, noting that a 360° scoring followed by complete Descemet stripping generally led to poorer results. In contrast, partial scoring over a few clock hours with Descemet removal via forceps was associated with successful corneal clearing and improved BCVA.^30^ Interestingly, our case deviates from these observations, as the 360° scoring and peeling still resulted in good long-term vision, suggesting that other, as yet undefined factors may also contribute to the success of DSO and merit further investigation to clarify the optimal approach.

DSO results also appear to be influenced by descemetorhexis size. Bleyen et al.^31^ observed that only 1 out of 8 patients with an 8-mm DSO achieved corneal clearance after 18 months, while Price et al. reported variable outcomes in three cases with a 6–6.5 mm descemetorhexis.^32^ In contrast, smaller diameters of 3–4 mm, as in our case, are associated with higher success rates.^28^ Nonetheless, the differing outcomes between the patient’s eyes underscore that while age, prior ocular surgeries, and potentially other patient-specific factors may play roles in achieving sustained visual improvement, the precise contributors to DSO’s success are not fully understood. This complexity suggests that outcomes may be influenced by an interplay of these elements rather than by any single variable alone.

Another factor currently under investigation to support endothelial recovery after DSO is the use of Rho kinase inhibitors (ROCKi). These agents are believed to aid endothelial cell migration and regeneration by potentially stimulating cell proliferation and reducing apoptosis. Multiple clinical trials are ongoing to evaluate ROCKi efficacy in promoting corneal clarity specifically after DSO procedures.^26,33–36^ If proven effective, ROCK inhibitors could become a valuable adjunct to DSO, particularly in cases with limited, central endothelial damage. This advancement could significantly enhance patient outcomes and further establish DSO as a viable alternative to DMEK, especially amidst increasing global shortages of donor tissue.^4^

Descemetectomy has several advantages compared to keratoplasty including the absence of a waiting time for a donor graft, no risk of graft immune rejection and no long-term postoperative steroid use. This is particularly relevant in patients with concomitant advanced glaucoma, such as in our case, as it eliminates the risk of steroid-induced ocular hypertension.^37^

This case offers several insights into the advantages but also the limitations of DSO. First, while DSO can provide sustained improvement, its effects may ‘expire’ over time, which is of course also true for DMEK-surgery itself.^38^ Second, even in cases of failure after DSO-procedure (early or late), successful visual rehabilitation can still be achieved with a secondary rescue DM graft. Third, the clinical observation of guttae after denuding the posterior stroma by means of DSO, as confirmed by specular microscopy images (Figures 3 and 4), supports the notion of de novo synthesis of Descemet membrane. Although histological confirmation is lacking, the imaging evidence strongly suggests endothelial regeneration and guttae formation post-DSO.

In conclusion, while endothelial keratoplasty remains the gold standard for the treatment of FED, DSO has potential as a temporary first-line treatment for patients with mild and localized endothelial changes. It can provide good visual acuity for several years; however, patients and surgeons must be aware that this benefit may be lost. In cases of early unsuccessful or late recurrent endothelial failure after DSO a DMEK can still be performed successfully.

## Supplemental Material

sj-pdf-1-oed-10.1177_25158414251359583 – Supplemental material for 10-Year outcome of descemet stripping only in a patient with Fuchs endothelial dystrophy: a case report Supplemental material, sj-pdf-1-oed-10.1177_25158414251359583 for 10-Year outcome of descemet stripping only in a patient with Fuchs endothelial dystrophy: a case report by Fuad Moayed, Friedrich Anton Steindor, Zaira Eleni Armeni, Markus Kohlhaas and Gerd Geerling in Therapeutic Advances in Ophthalmology

## Declarations

**Ethics approval and consent to participate:** This case report was exempted from full ethical review as it involves a single patient and no identifiable information is included. Written informed consent was obtained from the patient for participation in this case report.

**Consent for publication:** Written informed consent was obtained from the patient for publication of clinical information and images in this journal. The patient understands that their name and initials will not be published, and that efforts will be made to protect their identity, but anonymity cannot be fully guaranteed.

**Availability of data and materials:** The clinical data, including slit-lamp photographs and examination results, were collected as part of the case report. These data are available upon reasonable request from the corresponding author. Due to patient confidentiality and ethical guidelines, access will be provided in accordance with applicable regulations.
