# Supplementary material for: 10-Year outcome of descemet stripping only in a patient with Fuchs endothelial dystrophy: a case report
Source: Ther Adv Ophthalmol. 2025 Aug 30;17:25158414251359583. doi: 10.1177/25158414251359583 (PMC12398654; doi:10.1177/25158414251359583)
Supplement: sj-pdf-1-oed-10.1177_25158414251359583 – Supplemental material for 10-Year outcome of descemet stripping only in a patient with Fuchs endothelial dystrophy: a case report [file sj-pdf-1-oed-10.1177_25158414251359583.pdf]

CARE
case report guidelines

CARE Checklist of information to include when writing a case report

(cc) BY-NC-ND

Topic	Item	Checklist item description	Reported on Line
Title	1	The diagnosis or intervention of primary focus followed by the words "case report"	1
Key Words	2	2 to 5 key words that identify diagnoses or interventions in this case report, including "case report"	16
Abstract (no references)	3a	Introduction: What is unique about this case and what does it add to the scientific literature?	18-20, 34-39
	3b	Main symptoms and/or important clinical findings	21, 25-27, 29-33
	3c	The main diagnoses, therapeutic interventions, and outcomes	21-33
	3d	Conclusion—What is the main "take-away" lesson(s) from this case?	34-39
Introduction	4	One or two paragraphs summarizing why this case is unique (**may include references**)	74-79
Patient Information	5a	De-identified patient specific information.	81-86
	5b	Primary concerns and symptoms of the patient.	81-82
	5c	Medical, family, and psycho-social history including relevant genetic information	82-86
	5d	Relevant past interventions with outcomes	-
Clinical Findings	6	Describe significant physical examination (PE) and important clinical findings.	86-94
Timeline	7	Historical and current information from this episode of care organized as a timeline	127-182
Diagnostic Assessment	8a	Diagnostic testing (such as PE, laboratory testing, imaging, surveys).	86-94
	8b	Diagnostic challenges (such as access to testing, financial, or cultural)	-
	8c	Diagnosis (including other diagnoses considered)	82, 88-89, 93-94
	8d	Prognosis (such as staging in oncology) where applicable	-
Therapeutic Intervention	9a	Types of therapeutic intervention (such as pharmacologic, surgical, preventive, self-care)	95-99, 110-114, 119-123
	9b	Administration of therapeutic intervention (such as dosage, strength, duration)	-
	9c	Changes in therapeutic intervention (with rationale)	112-113, 119-120
Follow-up and Outcomes	10a	Clinician and patient-assessed outcomes (if available)	100, 103
	10b	Important follow-up diagnostic and other test results	106-120
	10c	Intervention adherence and tolerability (How was this assessed?)	113-126
	10d	Adverse and unanticipated events	110-114
Discussion	11a	A scientific discussion of the strengths AND limitations associated with this case report	187-194, 195-214, 225-240
	11b	Discussion of the relevant medical literature **with references**.	195-211, 216-224
	11c	The scientific rationale for any conclusions (including assessment of possible causes)	195-206, 216-224
	11d	The primary "take-away" lessons of this case report (without references) in a one paragraph conclusion	238-243
Patient Perspective	12	The patient should share their perspective in one to two paragraphs on the treatment(s) they received	-
Informed Consent	13	Did the patient give informed consent? Please provide if requested	Yes ☒ No ☐
